# Functional Differences in Visceral and Subcutaneous Fat Pads Originate from Differences in the Adipose Stem Cell

**DOI:** 10.1371/journal.pone.0036569

**Published:** 2012-05-04

**Authors:** Silvana Baglioni, Giulia Cantini, Giada Poli, Michela Francalanci, Roberta Squecco, Alessandra Di Franco, Elisa Borgogni, Salvatore Frontera, Gabriella Nesi, Francesco Liotta, Marcello Lucchese, Giuliano Perigli, Fabio Francini, Gianni Forti, Mario Serio, Michaela Luconi

**Affiliations:** 1 Endocrine Unit, Department of Clinical Physiopathology, University of Florence, Florence, Italy; 2 Department of Physiological Sciences, University of Florence, Florence, Italy; 3 Department of Human Pathology and Oncology, University of Florence, Florence, Italy; 4 Department of Internal Medicine, University of Florence, Florence, Italy; 5 General and Vascular Surgery, AOU Careggi, Florence, Italy; 6 Department of General Surgery, University of Florence, Florence, Italy; Pennington Biomedical Research Center, United States of America

## Abstract

Metabolic pathologies mainly originate from adipose tissue (AT) dysfunctions. AT differences are associated with fat-depot anatomic distribution in subcutaneous (SAT) and visceral omental (VAT) pads. We address the question whether the functional differences between the two compartments may be present early in the adipose stem cell (ASC) instead of being restricted to the mature adipocytes. Using a specific human ASC model, we evaluated proliferation/differentiation of ASC from abdominal SAT-(S-ASC) and VAT-(V-ASC) paired biopsies in parallel as well as the electrophysiological properties and functional activity of ASC and their *in vitro*-derived adipocytes. A dramatic difference in proliferation and adipogenic potential was observed between the two ASC populations, S-ASC having a growth rate and adipogenic potential significantly higher than V-ASC and giving rise to more functional and better organized adipocytes. To our knowledge, this is the first comprehensive electrophysiological analysis of ASC and derived-adipocytes, showing electrophysiological properties, such as membrane potential, capacitance and K^+^-current parameters which confirm the better functionality of S-ASC and their derived adipocytes. We document the greater ability of S-ASC-derived adipocytes to secrete adiponectin and their reduced susceptibility to lipolysis. These features may account for the metabolic differences observed between the SAT and VAT. Our findings suggest that VAT and SAT functional differences originate at the level of the adult ASC which maintains a memory of its fat pad of origin. Such stem cell differences may account for differential adipose depot susceptibility to the development of metabolic dysfunction and may represent a suitable target for specific therapeutic approaches.

## Introduction

Metabolic pathologies can originate from white adipose tissue dysfunctions. This organ can no longer be referred to as a mere energy storage site since it has been demonstrated to also display pivotal endocrine functions through the secretion of specific hormones called adipokines. Functional differences in the adipose tissue and the impact of its dysfunction on metabolism is associated with the regional distribution of fat depots, in particular subcutaneous (SAT) and visceral (VAT) adipose tissues [1]. Epidemiological studies highlighted that VAT accumulation associates with an increased metabolic risk and overall mortality [2], [3], whereas SAT expansion ameliorates insulin sensitivity and decreases type 2 diabetes risk [4]. Notably, in the mouse, SAT rather than VAT transplantation in either subcutaneous or visceral depots decreases circulating insulin and glucose levels, resulting in an improvement of glucose tolerance and insulin sensitivity [5], [6], suggesting that adipose tissue depots maintain an intrinsic memory of their site of origin.

Human adult adipose stem cell (ASC) populations have recently been characterized in parallel by paired biopsies from subcutaneous (S-ASC) and visceral omental (V-ASC) adipose tissue [7], representing a valuable cell model for the *in vitro* study of early events and processes occurring in the adipose tissue from different anatomical sites. Differences in gene expression and biological responses have been described in adipocytes and preadipocytes of the two adipose compartments [8]–[11]. In the present study, by the use of this specific model of human adult ASC cultures obtained from paired abdominal SAT and VAT biopsies, we address the question whether the functional differences observed between these tissues may be present early in the adipose stem cell which retains the memory of its fat pad of origin. In particular, we compare morphologic properties, proliferation activity, and adipogenic potential of the paired ASC populations and the functional properties of their *in vitro*-derived adipocytes. Previous electrophysiological studies have shown that preadipocytes and adipocytes express K^+^ channels [12], [13] and increasing evidence has pointed toward a functional relationship between resting membrane potential (RMP), K^+^ channel type expression and cell functions such as proliferation and differentiation [14]–[17]. The principal ion currents found in human bone marrow-derived mesenchymal stem cells (hBM-MSCs) [18], [19] and in ASC [7], [20] are two types of delayed-rectifier K^+^ currents (I_K,DR_): a noisy current showing a rapid activation/slow inactivation (I_Kr_) which probably corresponds to the Ca^2+^-activated, large conductance K^+^ current (I_BK_ or Maxi K^+^ current) and a slow activating K^+^ current named I_Ks_. The expression of the total K^+^ current of delayed rectifier (KDR) channels supporting these currents changes during the cell cycle and decreases during commitment and differentiation. In contrast, during these processes, there is a parallel increase in the size of the inward-rectifier K^+^ current (I_Kir_) and the cells become progressively more hyperpolarized [16]. The voltage threshold for I_KDR_ activation in stem cells is at a less depolarized potential compared to the resting membrane potential, RMP. This indicates that in ASC the RMP is maintained predominantly by the Na^+^/K^+^ ATP-ase pump and ion transporters with a small contribution of KDR channels. Conversely, in differentiating cells the progressive increased expression of Kir channels contributes to RMP maintenance. Notably, blockade of K^+^ channels reduces stem cell proliferation [13]. Here, we evaluated the modulation of the passive electrophysiological properties and K^+^ current types occurring in ASC during the process of differentiation as well as the putative differences characterizing ASC and the corresponding *in vitro*-derived adipocytes according to the adipose tissue of origin.

## Materials and Methods

### Ethics Statement

Adipose tissue samples were obtained, following written informed consent, from 18 subjects undergoing intra-abdominal laparoscopic surgery at AOU Careggi Hospital [7]. All the protocols have been approved by the Local Ethical Committee of AOU Careggi Hospital (CEL).

### Subjects

Adipose tissue samples were obtained from 18 subjects [9 females and 9 males: mean age ± SD: 60±18 yrs, 20–78 yrs; BMI 26.4±3.2 kg/m^2^, 20.2–29.5 kg/m^2^), undergoing intra-abdominal surgery. A total of 27 paired S-ASC and V-ASC populations have been obtained from stromal vascular fraction (SVF) of this cohort. We excluded all subjects affected by type 2 diabetes, cancer, infections, inflammation or taking thiazolidinediones or steroids.

### Isolation and culture of human adipose-derived stem cells from SAT and VAT

Cell isolation was performed as described [7] from adipose tissue biopsies obtained by laparoscopic surgery (4–6 gr). Briefly, under sterile conditions, adipose tissue samples were immediately placed in serum free (SF) Dulbecco's modified Eagle's medium (DMEM)/F12 supplemented with 200 µg/ml streptomycin and 200 U/ml penicillin. Adipose tissue biopsies were washed in PBS, then minced and digested with 1 mg/ml collagenase type I in PBS for 1 hour at 37°C in a shaking water bath. The pellet was collected by centrifugation at 650×*g* for 10 minutes and then treated with red blood cell lysis buffer (155 mM NH_4_Cl, 10 mM KHCO_3_, 0.1 mM EDTA) for 10 minutes at room temperature. After centrifugation, the cellular pellet was filtered through a 100 µm mesh filter to remove debris. The filtrate was centrifuged and the obtained stromal vascular fraction (SVF) was plated onto 100 mm cell culture dishes in complete culture medium (DMEM containing 20% fetal bovine serum (FBS), 100 µg/ml streptomycin, 100 U/ml penicillin, 2 mM L-glutamine, 1 µg/ml amphotericin-B). Cells were cultured at 37°C in humidified atmosphere with 5% CO_2_. After 24 hours non-adherent cells were removed and adherent cells were washed twice with PBS. Confluent cells were trypsinized and expanded in T75 flasks (passage 1, P1). A confluent and homogeneous fibroblast-like cell population was obtained after 2–3 weeks of cultures. In all the experiments, only cells at early culture passages were used (P1–P3) after an overnight serum lowering from 20 to 5%. Each experiment was performed at least 3 times.

### In vitro cell differentiation

For adipogenic differentiation, ASC were cultured in 10% FBS-DMEM, 0.5 mM 3-isobutyl-1-methylxanthine, 1 µM dexamethasone, 200 µM indomethacin and 10 µM insulin for 2 weeks, then shifted to 10% FBS-DMEM containing 1.7 µM insulin for another week [7], [21]. Adipogenic differentiation was demonstrated with Oil Red O or AdipoRed staining. For osteogenic differentiation, ASC were cultured in 10% FBS-DMEM, 0.1 µM dexamethasone, 50 µM ascorbate-2-phosphate, 10 mM β-glycerophosphate for 3 weeks [7], [21]. Osteogenic differentiation was demonstrated with Alizarin Red S staining.

### Cell dimension evaluation

Cell dimension analysis was performed by Scepter Handheld Automated Cell Counter (cat # PHCC00000, Millipore, Billerica, MA) in trypsinized cells resuspended at the appropriate concentration and obtained from 3 different subjects. Cell dimension distributions were generated by manually gating the different peaks and the cell concentrations recorded using Scepter Software 1.2. At least 2×10^5^ cells in duplicate were analyzed for each sample.

### ASC proliferation Assays

#### 5-Bromo-2′-deoxyuridine

A 5-bromo-2′-deoxyuridine (BrdU)-based ELISA kit (Roche Diagnostics, Mannheim, Germany) was used according to the manufacturer's protocol. Non confluent cells were incubated with 10 μM BrdU for 6 hours and absorbance was measured at 450 nm with a reference wavelength at 490 nm.

#### Cell count

Seeded cells were counted in haemocytometer every day. Mean cell number was obtained by counting 6 replicates for each point in each experiment. Dead cells were excluded by trypan blue staining.

#### MTS assay

Plated ASC were analyzed every day by MTS assay according to the manufacturer's instructions. Each experimental point was performed in five replicates in 6 independent experiments.

#### PD evaluation

Confluent ASC (passage 0) were counted in haemocytometer and re-plated at the same initial density. The number of population doublings (PD) was calculated as *n* of PD = log_2_ (N_i_/N_o_) (N_i_ = number of cells yielded; N_o_ = number of cells plated). Results were plotted according to the following equation: PD_(n)_ = PD_(n−1)_+PD_(n)_.

#### Ki-67

Immunocytochemical analysis with mouse anti-human Ki-67 antibody was performed on ethanol fixed/0.1% Triton-X permeabilized ASC with the Ventana Benchmark XT system (Ventana Medical Systems, Tucson, AZ). Ki-67 positive nuclei were counted on at least 100 cells. Negative controls were performed by omitting the primary antibody.

### RNA isolation and quantitative real time RT-PCR

RNA isolation and quantitative real time RT-PCR were performed as detailed elsewhere [7] using the primers/probes for the indicated genes (Applied Biosystems, Warrington, UK). The amount of target, normalized to GAPDH and relative to a calibrator, used as positive control, was given by 2^-ΔΔCt^ calculation.

### Lipid content quantification

The intracellular lipid content was measured by AdipoRed™ assay according to the manufacturer's instructions (Cambrex, MA). After 21 day differentiation, AdipoRed was added and fluorescence emission measured by 485/572 nm excitation/emission. Specific absorbance of differentiated adipocytes was calculated as fold increase on the non specific absorbance of the corresponding ASC samples in V- and S-populations. Each point was carried out in quadruplicate in 2 separate differentiation experiments performed with cells obtained from 5 different subjects.

### Lipolysis assay

*In vitro-*differentiated adipocytes treated with AdipoRed to stain intracellular lipids were incubated for additional 12 hours in insulin- and phenol red-free medium, in the presence or absence of 1 µM isoproterenol. Both isoproterenol-stimulated and basal lipolytic activity of S- and V-ADIPO was evaluated as the decrease in intracellular triglyceride content, and expressed as mean percentage of AdipoRed absorbance decrease normalized to the initial AdipoRed content, after subtracting the non specific ASC mean absorbance. Each point was carried out in quadruplicate in 2 separate differentiation experiments performed with cells obtained from 4 different subjects.

### Immunofluorescence cytochemistry

ASC grown on glass coverslips were subjected to adipose differentiation and fixation/permeabilization. Serum blocked coverslips were incubated with anti-FABP4 antibody (Santa Cruz Biotechnology, Heidelberg, Germany) followed by FITC-conjugated anti-rabbit IgG antibody. Fluorescence was acquired with a Leica DM4000 epifluorescence microscope (Leica Microsystems GmbH, Wetzlar, Germany). Negative controls (not shown) were performed avoiding primary antibodies.

### Western Blot Analysis

Thirty µg of proteins extracted from ASC, adipocytes and SAT/VAT, separated by reducing SDS-PAGE (10% and 15% acrylamide/bis acrylamide percentage for actin/adiponectin and FABP4 analysis, respectively) and transferred to PVDF membranes, were probed with primary antibodies (FABP4 and actin, Santa Cruz Biotechnology, Heidelberg, Germany; adiponectin, Vinci Biochem, Vinci, Italy) followed by peroxidase-secondary IgG. Image acquisition was performed with ChemiDoc XRS instrument (BIO-RAD Labs, Segrate, Italy).

### Adiponectin ELISA measurement

Confluent ASC were differentiated with the adipogenic medium. Conditioned media collected at 2 differentiation time points (14 and 21 days) were analyzed with DuoSet ELISA kit for adiponectin detection (R&D Systems, Minneapolis, MN). Each point was performed at least in quadruplicates on cells obtained from 4 different subjects.

### Flow Cytometry Analysis

Cell immunophenotypical analysis of ASC was performed by using the FITC-, PE- or APC-conjugated monoclonal antibodies against different surface antigens and their respective isotype controls (BD Biosciences, Mountain View, CA). At least 10^4^ cells were acquired from each sample and analyzed by flow cytometry (BDLSR II, BD Biosciences) using the BD DIVA and CellQuest software.

### Cytogenetic analysis of ASC

Preconfluent ASC were treated with Colcemid and hypotonic solution. Metaphases were prepared from cold ethanol/acetic acid (3∶1)–fixed cells staining slides with Trypsin-Giemsa (GTG-banded karyotype). At least 20 metaphase cells were analyzed. Results were scored by Chantal software equipped microscope (Leica Microsystems GmbH, Wetzlar, Germany).

### Electrophysiology

The electrophysiological properties of voltage-gated K^+^ currents of cultured V- and S-ASC, and V- and S-ADIPO were investigated on glass coverslip-adherent single cells by the whole-cell patch-clamp technique [7], using voltage-clamp and current-clamp modes. The electrode junction potential was evaluated before making the patch and was subtracted from the recorded intracellular potential. All experiments were carried in control physiological solution (150 mM NaCl, 5 mM KCl, 2.5 mM CaCl_2_, 1 mM MgCl_2_, 10 mM D-glucose and 10 mM HEPES) with the K^+^ channel blocker 4-aminopyridine, 4-AP (2 mM) or tetraethylammonium, TEA (20 mM), to avoid the occurrence of the transient outward potassium current I_to_ and with Nifedipine (10 µM) to block L-type Ca^2+^ current (I_Ca,L_). The standard solution to fill the electropipettes contained 100 mM potassium glutamate, 35 mM KCl, 5 mM MgCl_2_, 10 mM HEPES and 5 mM EGTA; pH was adjusted to 7.2 and had tip resistances of 2–3 MΩ. To record I_K,DR_ only, BaCl_2_ (0.4 mM) was added to the external solution to block I_Kir_ and to evaluate the presence of I_BK_ and I_Ks_, we used iberiotoxin, Ibtx (100 nM) and chromanol, Chr (50 µM), respectively. The RMP was evaluated in current-clamp mode. The delayed-rectifier K^+^ current (I_K,DR_) was recorded in voltage-clamp mode by applying voltage steps in 10 mV increments from −80 to50 mV with holding potential (HP) of −60 mV to block Na^+^ current (I_Na_) and T-type Ca^2+^ current (I_Ca,T_). Linear leak, voltage independent ionic currents and capacitive currents were withdrawn on-line using the P/4 procedure. To this end, the pClamp9 program applied 8 negative sub-pulses with voltage 4 fold lower than the test voltage at a HP of −60 mV in order to elicit only linear leak, voltage independent ionic currents and capacitive currents. The average of the currents generated by the sub-pulses were subtracted on-line from the test currents.

The occurrence of the Kir current was assessed in voltage-clamp by recording the currents in response to voltage ramps ranging from −120 to 50 mV over a period of 0.5 seconds, which were imposed every 1 minute from a holding potential of −60 mV and digitized at a rate of 5 kHz; two runs repeated every 20 seconds were averaged. First, we used the control physiological solution without Ba^2+^ (Control) to record any K^+^ current (I_K,DR_ and I_Kir_) followed by Ba^2+^ addition to block I_Kir_. Finally, the average of two ramps elicited in the presence of Ba^2+^ was subtracted from control currents to evaluate I_Kir_.

The C_m_ value was considered as an index of the cell surface area assuming that membrane-specific capacitance is constant at 1 μF/cm^2^. To allow comparison of test current recorded from different cells, the resting membrane conductance, G_m_, was normalized to C_m_, G_m_/C_m_ (in nS/pF). The current amplitudes (I) were normalized to cell linear capacitance (C_m_) to allow comparison of test currents recorded from different cells. The ratio I/C_m_ is then proposed as current density. For RMP evaluation the small (2–5 mV) liquid junction potential was corrected. The patch pipette was connected to a micromanipulator (Narishige International USA) and an Axopatch 200B amplifier (Axon Instruments, USA). Voltage-clamp protocol generation and data acquisition were controlled by using an output and an input of the A/D−D/A interfaces (Digidata 1200; Axon Instruments, USA) and pClamp9 software (Axon Instruments, USA).

### Statistical Analysis

Statistical analysis was performed using SPSS 17.0 software (SPSS Inc. Chicago, IL, USA). The Kolmogorov–Smirnov test was used to verify data normal distribution. Student's *t* test was applied for statistical analysis of two classes of data. Non parametric distributions were analyzed using Wilcoxon test. A P<0.05 was considered significant. Data are expressed as mean ± SE or elsewhere indicated.

## Results

### Differences in cell proliferation

**Figure 1:**
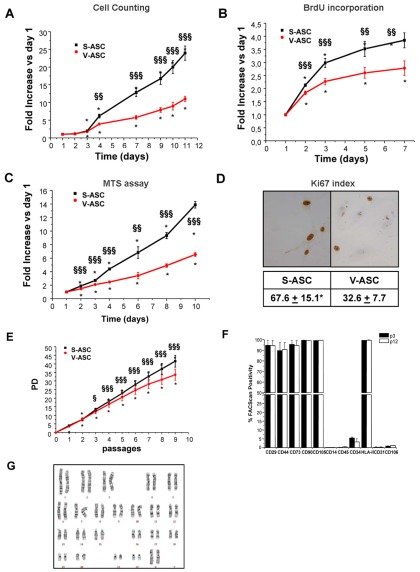
Different proliferation rate in S-ASC and V-ASC. Growth curves were obtained for each population by haemocytometer cell counting (A), evaluation of BrdU incorporation (B), and MTS assay (C) at each time point. Results are expressed as mean ± SE fold increase of cell counts (A), BrdU (B) and MTS absorbance (C) at each time point over day 1 in at least 4 ASC populations derived from at least 4 independent subjects. *P<0.001 versus respective day 1; §§P<0.01, §§§P<0.001 S- versus V-ASC at the corresponding time point. (D) Ki-67 proliferation index is calculated as mean ± SE percentage of positive cells counted in at least 20 fields for each slide. 4 ASC populations were obtained from 4 different subjects, *P<0.001 versus the corresponding V-ASC population. (E) Population doubling (PD) curves were obtained by cell counting at different passages of S-ASC and V-ASC cultures expanded for about 2 months. Results are expressed as mean ± SE PD numbers obtained by counting cells in triplicates from 3 independent subjects; *P<0.001, versus respective passage 0; §P<0.05, §§§P<0.001 versus the corresponding V-ASC. (F) Immunophenotype of V-ASC obtained at 2 different passages (P3 and 12). Data correspond to mean ± SE FACScan percentage of positive cells for the indicated surface markers previously demonstrated to characterize ASC populations (7); cells were obtained from n = 2 independent subjects. Similar data were obtained for the corresponding S-ASC at the same passage. No statistically significant differences have been observed in the expression of the indicated markers between the two passages. (G) Representative karyotype analysis of V-ASC at passage 12, corresponding to about 3 months of culture. Similar data has been obtained for the corresponding S-ASC at the same passage, in 2 different subjects. Karyotype analysis was performed on ASC obtained from 2 different subjects.

When the ability of ASC to proliferate *in vitro* was evaluated, a statistically significant difference in the proliferation rate between the two paired populations was observed. In particular, the growth rate was significantly higher in S-ASC than in V-ASC, as evaluated by cell counting (Fig. 1A) and bromodeoxyuridine incorporation (Fig. 1B) and confirmed by Ki-67 immunostaining (Fig. 1D). Moreover, the metabolic profile, evaluated by the MTS method, was significantly different between the two populations, with S-ASC being more active than V-ASC (Fig. 1C). Population doubling calculated over 2 months of cell growth showed a significant diversion of the two curves starting from passage 3 (each passage corresponding to 1 week of culture, Fig. 1E). In order to exclude any potential transforming effect on ASC populations due to long term *in vitro* culture, we analyzed both cell immunophenotype and karyotype in long culture populations. Compared to early passage (P3), no significant alteration in cell immunophenotype was detectable in the same ASC population cultured up to P12 (Fig. 1F). The normal cell karyotype of this P12 population is shown in Fig. 1G.

**Figure 2:**
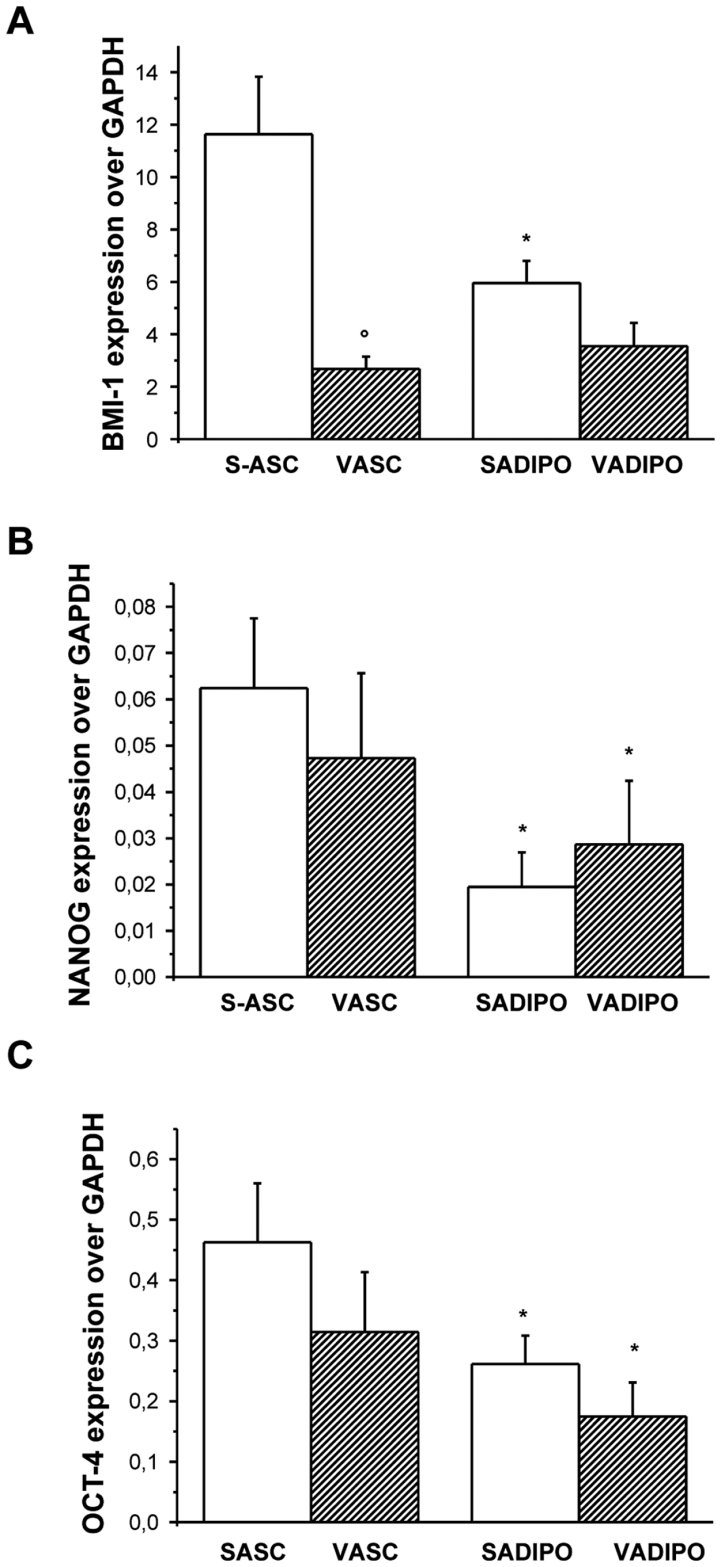
Stemness marker expression in S-ASC and V-ASC. Quantitative Real Time RT-PCR was performed on mRNA extracted from both S- and V-ASC and from the corresponding *in vitro*-differentiated adipocytes, to evaluate BMI-1, (A, n = 10 subjects) NANOG (B, n = 5 subjects) and OCT-4 (C, n = 5 subjects) expression. Data are expressed as the mean±SE gene expression versus the housekeeping GAPDH gene. °P<0.001 S- versus V-ASC and *P<0.05 adipocytes versus respective ASC.

The *in vitro* and *in vivo* maintenance of progenitor and stem cell activity mainly depends on the expression of the polycomb gene *bmi-1* [22], [23]. Quantitative real time RT-PCR of BMI-1 expression (Fig. 2A) showed that S-ASC exhibits a significantly higher expression of this polycomb gene which is involved in the regulation of cell cycle and senescence in the haematopoietic compartment [24]. Conversely, no difference in the expression of the two embryonic stemness markers NANOG (Fig. 2B) and OCT-4 (Fig. 2C) was detectable in ASC, suggesting a similar undifferentiated state between the 2 populations. Interestingly, *in vitro*-induced adipogenic differentiation was accompanied by a decrease in NANOG (Fig. 2B) and OCT-4 (Fig. 2C) expression, while BMI-1 (Fig. 2A) significantly decreased in adipocytes derived from S-ASC only, remaining constant in induced-V-ASC.

No expression of human telomere reverse transcriptase (hTERT) associated with telomearase activity was detectable in either S- or V-ASC (not shown).

### Differences in differentiation potential

**Figure 3:**
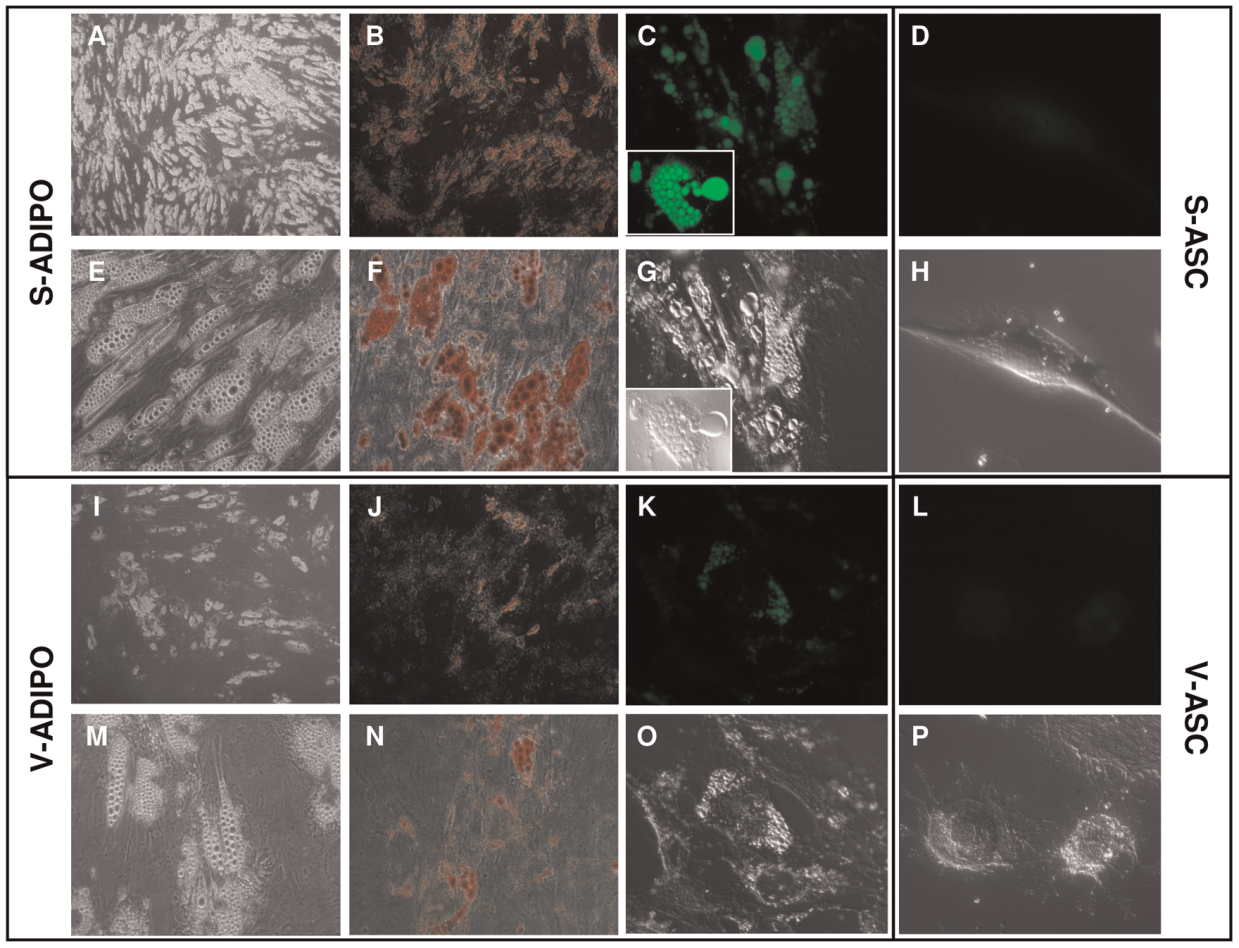
Staining of intracellular triglyceride depots in adipocytes obtained from *in vitro* differentiation of S- and V-ASC. Dark (A,I; 5× magnification) and bright field (E,M, G,O,H,P; 40× magnification) microscopy, ORO staining of neutral lipids (B,J; 5× magnification; F,N; 40× magnification) and fluorescence microscopy (40× magnification) of *in vitro*-differentiated S- (C) and V- (K) adipocytes and of the corresponding S- (D) and V- (L) ASC stained with AdipoRed evidentiate a qualitative higher number of lipid droplets and a higher number of differentiated adipocytes in the S- compared to V- populations. No staining was present in ASC, confirming the high specificity of AdipoRed binding to triglycerides. Representative of cell populations obtained at least from n = 4 different subjects.

BMI-1 expression differences between V-ASC and S-ASC prompted us to investigate ASC differentiation potential. Previously, we demonstrated that V-ASC and S-ASC cultures *in vitro* are able to differentiate towards specific lineages when maintained in the appropriate inductive media [7], confirming the multipotency of these stem populations. Following 3 weeks of *in vitro*-induced adipogenic differentiation, dark (Fig 3A,I) and bright (Fig 3E,M) field microscopy as well as Oil Red O staining (Fig 3B,F,J,N) and AdipoRed fluorescence microscopy (Fig 3C,D,K,L) showed a qualitatively higher number of well differentiated adipocytes in induced S-ASC compared to induced V-ASC, which also presented smaller and less well defined intracellular lipid droplets.

**Figure 4:**
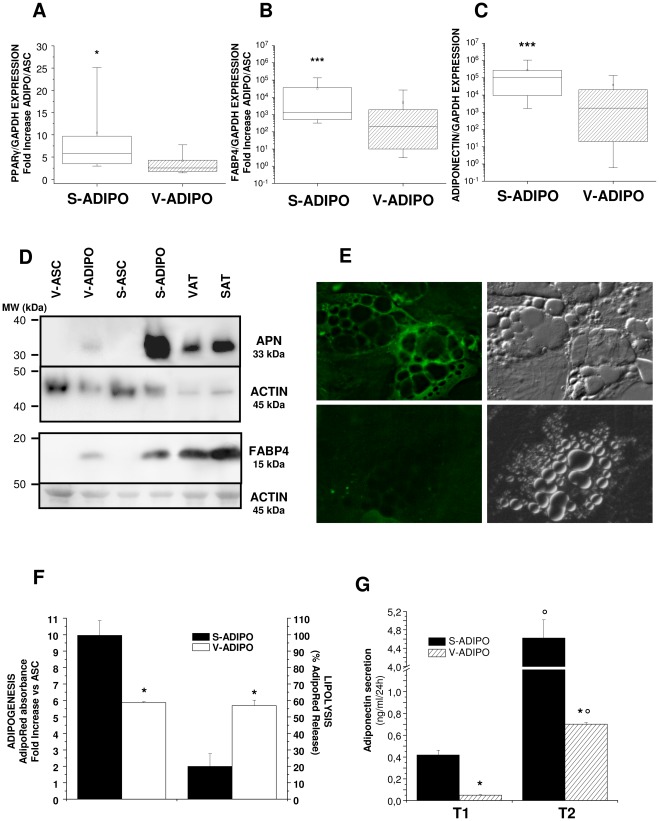
Differences in adipogenic potential and functional capabilities in ASC and in their *in vitro*-derived adipocytes. Expression of the adipogenic genes PPARgamma (A), FABP4 (B) and adiponectin (C) evaluated by quantitative real time RT-PCR is higher in S- compared to derived V-ADIPO. Data are expressed by box charts of gene expression ratio versus GAPDH (C) or fold increase between adipocytes and ASC (A, B). Boxes indicate the 25^th^ (lower) and 75^th^ (upper) percentiles. Horizontal lines and dots in the boxes indicate the 50^th^ percentile value (median) and mean value, respectively. Vertical lines give the 10^th^ and 90^th^ percentile limits of the data. Statistical analysis for non-parametric distribution was performed with Wilcoxon text: *P<0.05, ***P<0.001 S- versus V-ADIPO, n = 30 experiments with cells obtained from 14 independent subjects. D: Western Blot analysis of Adiponectin (upper panel) and FABP4 (lower panel) protein expression in ASC compared to the derived adipocytes. Molecular weight (MW) in kDa has been indicated for both standards and proteins of interest. Equal protein loading was verified by probing for the housekeeping protein actin. SAT and VAT samples from the same subject were run as positive controls. Representative of 5 independent experiments performed on cells obtained from 5 subjects. E: Immunofluorescence analysis of FABP4 (left panel) in *in vitro*-differentiated S-ADIPO (upper panel) and V-ADIPO (lower panel) revealed positivity for the enzyme around the intracellular lipid droplets. Right panels: corresponding bright field microscopy. F: Adipogenic potential of S- and V-ASC has been evaluated as AdipoRed staining of intracellular lipid droplets in the derived adipocytes. Results are expressed as mean ± SE fold increase of AdipoRed absorbance (left axis) in adipocytes versus the corresponding ASC. Lipolytic activity (right axis) of the same adipocytes evaluated as AdipoRed absorbance fold decrease following 12 h treatment with 1 µM isoproterenol. *P<0.001 S- versus V-ADIPO. G: Adiponectin secretion evaluated during *in vitro*-induced adipogenesis in S- and V-ASC at two different time points (T1 and T2, 14 and 21 days of differentiation respectively) by ELISA adiponectin kit. °P<0.001 T2 versus T1; *P<0.001 S- versus V-ADIPO.

Expression of early genes involved in adipocyte differentiation, such as PPARgamma, revealed a statistically significant higher expression in adipocytes derived from S-ASC compared to V-ASC (Fig. 4A). Similar results have been obtained for late differentiation markers such as FABP4 (Fig. 4B) and adiponectin (Fig. 4C). Western Blot analysis of cell lysates confirmed that adipocytes induced from S-ASC synthesize more adiponectin (Fig. 4D, upper panel) and FABP4 (Fig. 4D, lower panel). This latter protein, which is involved in fatty acid transport inside the cell, is functionally located around intracellular lipid droplets, as shown by immunocytochemistry of *in vitro*-differentiated adipocytes from S-ASC (Fig. 4E, upper panel) and V-ASC (Fig. 4E, lower panel). To further confirm that S-ASC possess a higher adipogenic potential, we evaluated the ability of both V-ASC and S-ASC to accumulate triglycerides intracellularly during *in vitro*-induced adipogenesis. Quantitative AdipoRed staining confirmed a statistically significant higher level of intracellular lipid accumulation in adipocyte populations differentiated from S-ASC versus V-ASC (Fig. 4F). Conversely, a significantly higher β-adrenergic lipolytic activity was detectable in differentiated V-ADIPO populations compared to the corresponding S-ADIPO, as measured by AdipoRed staining of the remaining intracellular triglycerides following 12 hour treatment with 1 µM isoproterenol (Fig. 4F). Finally, we measured the ability of *in vitro*-differentiated adipocytes to actively secrete adiponectin as a marker of functional differentiation. Adiponectin concentrations were significantly higher in conditioned media of S-ADIPO compared to V-ADIPO at both differentiation time points (Fig. 4G). Moreover, adiponectin secretion time-dependently increased in both populations, but in particular in S-ADIPO, according to the level of adipocyte maturity, supporting the conclusion that the *in vitro* differentiation process functionally resembles *in vivo* physiological adipogenesis (Fig. 4G).

According to their multipotent nature, ASC cells are able to *in vitro* differentiate toward the osteogenic phenotype when properly stimulated with the appropriate differentiation medium [7]. In order to quantify any putative difference also in the osteogenic potential of the two ASC populations, their osteoblastogenic ability was assessed following 3 weeks of *in vitro* specific differentiation. S-ASC showed a significantly higher expression of the osteogenic marker RUNX2 compared to V-ASC (mean ± SE RUNX2 fold increase on GAPDH: 27.5±13.0 vs 4.4±0.5, P<0.05, n = 7 populations from 4 subject), confirming a higher osteogenic efficiency rate for S-ASC.

### Electrophysiological properties of ASC and in vitro-differentiated adipocytes

**Figure 5:**
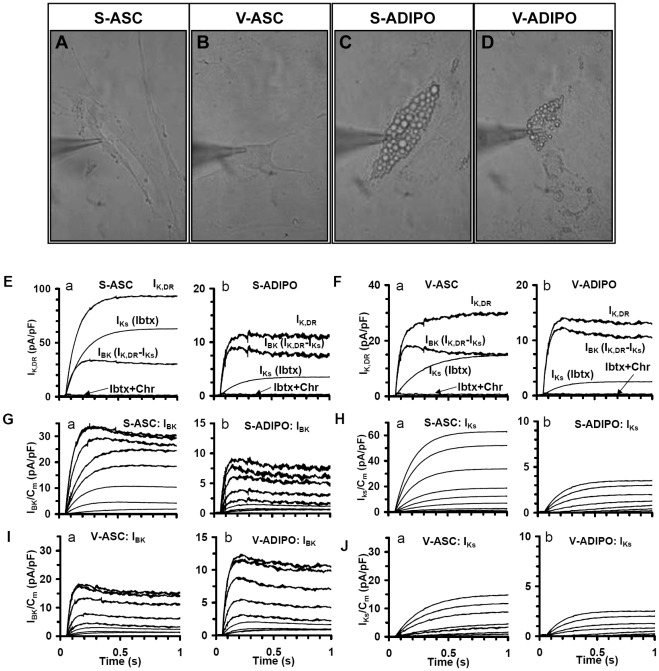
ASC and *in vitro*-differentiated derived adipocytes express two types of delayed rectifier K^+^ currents. Light microscopy of a S-ASC (A), V-ASC (B), S-ADIPO (C) and V-ADIPO (D) plated on glass coverslip and impaled by the patch pipette for electrophysiological records (magnification 40X). (E–F) Typical I_K,DR_ trace currents elicited by a voltage step to 50 mV from a holding potential of −60 mV in Control solution with 4-AP (2 mM), Nifedipine (10 µM) and Ba^2+^(0.1 mM) in S-ASC (Ea), S-ADIPO (Eb), V-ASC (Fa) and V-ADIPO (Fb). In each cell type, I_Ks_ traces are obtained in the presence of Ibtx (100 nM) and I_BK_ traces by subtracting I_Ks_ from I_K,DR_. By adding Chr (50 µM) only a very small residual current was recorded (Ibtx+Chr current traces). (G–J) Family of I_BK_ (G and I) and I_Ks_ (H and J) family of current traces evaluated as in panels E and F (same cells) from pharmacological dissection with voltage steps of 10 mV increments (from −80 to 50 mV; HP of −60 mV. Note the different ordinate scale in all panels.

Next, we performed a detailed analysis of the electrophysiological properties of ASC and of the derived *in vitro*-differentiated adipocytes by patch clamp techniques (Fig. 5A–D), which further confirmed functional differences in the two stem and differentiated populations.

The RMP, evaluated by the whole cell patch clamp technique in current-clamp condition, was recorded from V- and S-ASC and from the corresponding *in vitro*-differentiated adipocytes (V- and S-ADIPO). RMP was significantly more hyperpolarized in S-ASC and S-ADIPO than in V-ASC and V-ADIPO, respectively (Table 1), suggesting that S-ASC were more prone than V-ASC to differentiate towards the adipose phenotype [16].

**Table 1:** Electrophysiological properties of ASC and of the derived *in vitro*-differentiated adipocytes.

	UNITS	S-ASC	V-ASC	S-ADIPO	V-ADIPO
**RMP**	mV	−50.1±5.3	−45.6±4.3*	−52.4±5.6	−43.1±4.8**
**C_m_**	pF	12.0±1.2	19.1±1.6**	27.3±2.2^§§^	16.2±1.6**^§§^
**G_m_**	nS	31.2±2.8	45.6±4.1**	24.5±2.2	30.4±2.8**^§§^
**G_m_/C_m_**	nS/pF	2.6±0.2	2.4±0.2	0.9±0.1^§^	1.9±0.2**^§^
**V_th_ (I_Ks_)**	mV	−24.3±2.7	−12.4±2.6***	−12.3± 2.9^§§§^	−10.3±2.7
**V_th_ (I_BK_)**	mV	−22.1±2.9	−12.2±2.1***	−32.8± 3.0^§§§^	−32.6±2.5^§§§^
**V_th_ (I_Kir_)**	mV	−72.1±7.1	−69.2±8.1	−72.4±6.8	−72.5±7.4
**τ (I_Ks_)**	ms	160.5±15.1	220.5± 19.2***	180.5± 14.6^§^	200.8± 18.4***^§^
**τ (I_BK_)**	ms	78.3±7.6	36.4±6.3***	40.3±5.6^§§^	50.2±6.3*^§§^
**I_Ks_/C_m_**	pA/pF	60.7±5.5	14.2±1.3***	4.2±0.4^§§§^	2.5±0.2**^§§§^
**I_BK_/C_m_**	pA/pF	33.6±3.0	17.9±1.6**	8.0±0.7^§§§^	11.3±1.0**^§§^
**I_Kir_/C_m_**	pA/pF	−10.2±1.3	−9.8±1.0	−101± 10.2^§§§^	−59± 7.1***^§§§^

Electrophysiological analysis was performed on ASC and their corresponding *in vitro*-differentiated adipocytes in patch clamp condition. RMP: resting membrane potential. C_m_: membrane capacitance. G_m_ and G_m_/C_m_: total and specific membrane conductance, respectively. V_th_: voltage threshold of activation of the three types of K^+^ currents investigated (I_BK_, I_Ks_ and I_Kir_). K^+^ current activation time constant (τ) for I_BK_ and I_Ks_ and current density I_BK_/C_m_ and I_Ks_/C_m_ values have been obtained at 50 mV and I_Kir_/C_m_ at −110 mV. Mean ±SE values are reported. Student's *t* test was applied to compare paired samples: **,***P<0.01 and 0.001 V- versus S-ASC and V- versus S-ADIPO; §, §§, §§§ P<0.05, 0.01, 0.001 S- or V-ADIPO versus the corresponding ASC. Total number of analyzed cells (n) in populations obtained from 5 different subjects were: S-ASC (n = 84), V-ASC (n = 82), S-ADIPO (n = 54), V-ADIPO (n = 52); for I_Kir_: S-ASC (n = 17), V-ASC (n = 18), S-ADIPO (n = 28), V-ADIPO (n = 24). mV: milli Volt; pF: pico Faraday; nS: nano Siemens; ms: milli seconds; pA: pico Ampere.

The whole cell patch clamp technique in voltage clamp mode was used to study the passive properties of both stem and differentiated populations. Electrophysiological measurement of ASC linear capacitance (C_m_), which is an indirect index of the cell surface area, provided evidence that V-ASC were larger than S-ASC (Table 1). Dimensional analysis performed by Scepter Handheld Automated Cell Counter (Millipore) on trypsinized ASC, confirmed the significantly greater diameter observed in V-ASC (mean cell diameter: 23.4±4.8 µm V-ASC and 20.5±3.9 µm S-ASC, P<0.005, n = 3 cell populations from 3 different subjects). Adipocytes differentiated from S-ASC increased their C_m_ and surface compared to the stem cell, in accordance with the functional development of the mature cell (Table 1). Conversely, *in vitro*-differentiated adipocytes derived from V-ASC displayed a significantly lower C_m_ compared to the corresponding ASC and were significantly smaller than the corresponding S-ADIPO (Table 1), suggesting that the latter undergo a more functional differentiation process accompanied by a better organized accumulation of intracellular triglycerides. The G_m_ conductance, an index of resting cell permeability, was higher in V-ASC than in S-ASC, and the same difference was observed upon differentiation, where G_m_ values were still significantly higher in V- than S-ADIPO (Table 1). The specific cell permeability (G_m_/C_m_) showed an opposite trend, being reduced in ADIPO with respect to ASC. Such changes were greater in S-ADIPO than in V-ADIPO (65% versus 20% decrease; Table 1). The resting conductance density is related to specific membrane leak and its reduced value is an index of an improved resting membrane obstacle to passive ions fluxes. Thus, the total membrane conductance, G_m_, increases with the C_m_ increase but the relative increase is lower if G_m_/C_m_ is small. This is the case of S-ADIPO respect to V-ADIPO. Accordingly, the G_m_ value is smaller in the larger S-ADIPO than in the V-ADIPO. Again, this supports a greater differentiation potential of S- compared to V-ASC.

**Figure 6:**
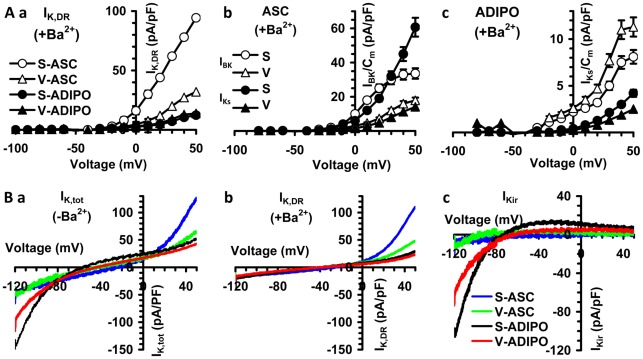
Voltage dependence of the two kind of I_K,DR_ (I_BK_ and I_Ks_) and of I_Kir_. (A) Plots represent the maximum mean value of I_K,DR_ (a), I_BK_ and I_Ks_ (b,c) as a function of the applied voltage step recorded in the presence of Ba^2+^ from all experiments as in Fig. 6 G–J. Note the different ordinate scale for ASC and ADIPO. Panels put in evidence that I_K,DR_, I_BK_ and I_Ks_ are reduced in size in the ADIPO versus the corresponding ASC. (B) K^+^ currents in representative cells elicited by voltage ramp stimulation in Control external solution without Ba^2+^ recording the total K^+^ currents (I_K,tot_) (a, – Ba^2+^) and after adding 0.1 mM Ba^2+^ to block I_Kir_ and record I_K,DR_ (b, +Ba^2+^ 0.1 mM). I_Kir_ have been obtained by detracting current traces recorded in the presence of Ba^2+^ from those in the absence (c). I_K_/C_m_ and V_th_ mean ± SE values and number of experiments are indicated in Tab 2.

Both ASC and *in vitro*-derived adipocytes were then assessed for the functional expression of specific K^+^ currents. Electrophysiological analysis confirmed the mesenchymal nature of these cells, since in Control solution without nifedipine they did not show Na^+^ and Ca^2+^ currents but only outwards currents. As these currents were unaffected by Ba^2+^ and blocked by 20 mM-TEA or 2 mM 4-AP solution (not shown), they can be reasonably assumed to be I_K,DR_. These currents could be pharmacologically dissected into two different kinds of outward K^+^ currents (Fig. 5Ea and Fa) observed in stem cells [7], [18]–[20]: 1) a slowly activating current, I_Ks_, Chr-sensitive; 2) a noisy rapidly activating and slowly inactivating current that saturated at positive potentials, I_BK_, Ibtx-sensitive [18]. In particular, in each cell type, the family of I_Ks_ traces was obtained in the presence of Ibtx (100 nM) (Fig. 5Ha and Ja) and I_BK_ traces were determined by subtracting I_Ks_ from I_K,DR_ (Fig. 6Ga and Ia). After adding Chr (50 µM) only small residual currents were recorded (Ibtx + Chr current traces).

In S-ASC, IBK and IKs had a more negative voltage threshold and a greater size with respect to V-ASC (Fig. 5Ga and Ha versus 5Ia and Ja, respectively; Fig. 6B; Tab. 1), resulting in a more hyperpolarized resting membrane potential and a better control of the membrane potential depolarization induced by stimuli such as hormones in S- than in V-ASC. Following adipogenic differentiation, both I_Ks_ and I_BK_ drastically decreased in size (Fig. 5 E–J and 7Ac; Table 1) and, in both V- and S-ADIPO, I_BK_ showed a greater size than I_Ks_. The voltage threshold of I_BK_ (V_th_) was shifted towards more negative potentials, whereas the opposite occurred for I_Ks_ (Fig. 6A and Table 1). Thus, upon differentiation the slowly activating I_Ks_ observed as predominant in S-ASC, was replaced by the rapid I_BK_ activated at more negative potentials. Again, major differences were observed in adipocytes from S- than V-ASC, further indicating the greater plasticity/differentiation potential of S-ASC. The changes between S- and V- populations observed during adipogenesis in these two types of K+ currents involved not only the voltage threshold and current size but also the current activation time constant (τ), (Table 1).

The application of 500 mseconds voltage ramp from −120 to 50 mV (holding potential of −60 mV) evoked both inward and outward currents in S- and V-ADIPO (Fig. 6Ba). Upon application of the K_ir_ channel blocker Ba^2+^ (0.1 mM), currents activated in the inward direction by voltages negative to −30 mV were inhibited and only residual linear leak currents could be observed (Fig. 6Bb). In contrast, outward currents evoked by voltages positive to −30 mV (I_K,DR_) were essentially unaffected by the same concentration of Ba^2+^ (Fig. 6Bb). Such effects were fully reversible upon Ba^2+^ washout. The Ba^2+^-sensitive current, evaluated by detracting the current recorded in the presence of Ba^2+^ from control, revealed the characteristics of inward rectification, having a reversal potential of −75.4±5.8 (V-ADIPO; n = 24) and −78.2±6.2 mV (S-ADIPO; n = 28) in a 5 to 135 mM K^+^ gradient (Fig. 6Bc). These values were similar to the calculated K^+^ equilibrium potential under our experimental conditions of −82 mV. The I_Kir_ was greater in S- than in V-ADIPO and, notably, did not show a complete rectification at more positive potentials, particularly in S-ADIPO. In contrast, I_Kir_ in S- (n = 17) and V-ASC (n = 18) were very small in size (Fig. 6Bc; Table 1).

## Discussion

White adipose tissue shows different properties depending on the anatomical distribution of the adipose tissue [1], [3], [25]. The differential physiological effects and metabolic risk due to expansion of visceral (VAT) rather than subcutaneous (SAT) adipose tissue have been well documented in epidemiological and physiological studies [4], [26]–[28] and may be based on intrinsic differences in the differentiated adipose cell [29]–[36].

In the present study, we demonstrate that the differences observed in mature adipocytes and adipose tissues are related to the adult stem cells. In contrast to previous studies addressing the properties of subcutaneous and visceral preadipocytes [8]-[10], [32], [33], [35], which might be already committed to some extent, our results are based on a specific approach that compares the properties of adult stem cells, immunophenotyped for mesenchymal and stem properties, obtained from paired abdominal adipose biopsies of the same subjects (ruling out individual genetic variability, 7] and of the *in vitro*-differentiated adipocytes. Adult adipose stem cells have been well characterized for their mesenchymal origin and share properties very similar to bone marrow derived mesenchymal stem cells [7], [12], [37]–[41]. In particular, such subcutaneous stem cell population displayed high self-renewal ability, very low tumorigenic potential and were able to differentiate into various lineages including adipocytes and osteoblasts as well as to support *in vivo* regenerative processes [37]–[39]. The development of reliable human stem cell models for evaluating the molecular events underlying adipogenesis is mandatory, as different regulatory mechanisms in the adipogenic process have been described between rodents and humans [42]. In our previous work, we reported the isolation and characterization of the two adult adipose stem cell populations obtained from subcutaneous and visceral adipose tissue [7]. In that paper, we had focused on the novel V-ASC population and only qualitatively compared S- and V-ASC *in vitro* differentiation towards the adipose phenotype. In the present paper, we further characterized and quantitatively compared the proliferation and differentiation abilities of the two stem populations as well as of their *in vitro* derived adipocytes. This analysis was obtained by performing the experiments in parallel conditions, enabling us to fully and strictly compare the properties of the stem cells and of their corresponding *in vitro* derived adipocytes. S-ASC showed a higher *in vitro* proliferation rate, which becomes evident as early as the first days of culture. These results are supported by a significant difference in the population doubling in long term culture. The occurrence of any potential cell transformation has been ruled out by cell karyotyping and immunophenotyping, confirming the absence of transformation and tumorigenesis reported in human adipose tissue-derived mesenchymal stem cells transplanted in nude mice [39], [43], [44]. The reported immunophenotype of both S- and V-ASC is comparable to that already reported for these cells in our previous work [7]. The higher proliferative ability of S-ASC may also provide an intriguing support to the adipose tissue expandability hypothesis underlying the pathogenesis of metabolic diseases. According to this hypothesis, adipose tissue expansion is regarded as a buffering response of the organism to store excess nutrients and lipids [25], [45]. In agreement with the high proliferative and lipogenic/adipogenic ability of ASC in SAT, subcutaneous rather than visceral fat may expand in response to high fat diet to accomodate excess levels of potentially toxic free fatty acids [25], [45]. In obese subjects, when SAT is no longer able to maintain its role as an energy “sink” by its continued expansion, the lipids accumulate in other tissues. Such ectopic lipid deposition can cause insulin resistance, cardiovascular complications and other lipotoxic effects [45]. Based on our findings, it may also be hypothesized that a differential regional mechanism of fat mass expansion under the metabolic dysregulation pressure characterizing obesity development, being SAT more capable of hyperplasia and VAT of hypertrophy. Although under healthy conditions, adipocytes in SAT are bigger, better organized and functional than the corresponding ones in VAT, under metabolic stress, VAT responds with a deranged expansion associated with hypertrophy rather than hyperplasia. In particular, in epidemiologic studies, subjects with adipose hypertrophy have a significantly lower number of adipocytes than those with hyperplasia [46]. Hyperplasia is a less deleterious mechanism of fat expansion in which the adipocytes are still functional, whereas the hypertrophic adipocytes are cells prone to inflammation, apoptosis, fibrosis and release of free fatty acids (FFA). Rapid turnover of cells in the adipocyte population has recently been demonstrated in murine adipose tissue [47]. The expression of the two embryonic stem cell markers found in adult stem cells [48], *nanog* and *oct-4*, did not differed between S- and V-ASC and was significantly higher than that of the corresponding adipocytes, indicating a similar undifferentiated state of the 2 stem cell populations. In contrast, *bmi-1* gene expression was more robust in S- compared to V-ASC. Self-renewal potential of stem cells has been described to be maintained not only in the haematopoietic lineage [24] but also in neural stem [49] and in cancer cells [50] by the expression of the polycomb gene *bmi-1* [22], [23]. In addition, another polycomb gene member, *ezh2*, has recently been demonstrated to regulate adipogenesis through repression of the Wnt/β catenin pathway [51], which is also negatively controlled by the stem gene *oct-4* [52]. Our findings provide intriguing evidence that, although showing a similar undifferentiated state, S-ASC have greater self-renewal and differentiation potential as compared to V-ASC. Accordingly, adipocytes from S-ASC are more numerous, better shaped and express higher levels of adipogenic genes and proteins characterizing the mature and functional cell. In particular, S-ADIPO display enhanced functionality compared to the corresponding V-ADIPO, showing not only a greater ability to accumulate intracellular triglycerides in a higher number of larger lipid droplets, but also displaying a significantly higher adiponectin secretory ability. Conversely, *in vitro*-differentiated V-ADIPO are more sensitive to β-adrenergic lipolytic stimuli, consistent with previous publications [53], [54]. Interestingly, an increased VAT contribution to FFA release, resulting in hepatic lipid accumulation and the development of insulin resistance, has been associated with obesity [55]. Moreover, previous studies suggest that VAT and its adipocytes display receptors with lower affinity for insulin both in lean and obese subjects [56] and that in this depot the intracellular signaling cascade in response to insulin is less sensitive than in SAT [11], [30]. In light of our findings, regional differences originating early at the level of adipose stem cells are conferred to the derived adipocytes. As a consequence, VAT rather than SAT displays lower sensitivity to the lipogenic and anti-lipolytic effects of insulin and higher sensitivity to β-adrenergic stimuli. The *in vitro*-differentiated adipocytes from ASC are functionally mature cells, capable not only of lipid storage but also to respond to lipolytic stimuli and to secrete adiponectin. In particular, adiponectin levels in conditioned media increase accordingly with the degree of adipocytes maturation. S-ASC and V-ASC not only differ in the levels of adiponectin expression but in their ability to functionally secrete this specific adipokine. The reduced rate of adiponectin secretion in V- compared to S- ADIPO reflects functional differences of V- versus S-ASC, possibly due to differences in adiponectin processing. Our data provide intriguing explanations for the metabolic profile improvement observed in mice transplanted with subcutaneous but not with visceral adipose tissue, independently of the site of transplantation [5], [57]. This effect is maintained over time, suggesting that newly differentiated adipocytes in transplanted tissue derive their memory from the adipose depot of origin.

The electrophysiological experiments confirm the stem cell nature of S- and V-ASC, since they both show two kinds of I_K,DR_ (I_BK_ and I_Ks_) similar to those previously recorded in mesenchymal stem cells [7], [15], [16], [18]–[20] whereas the I_Kir_ are less well expressed. However, these patch clamp studies provide evidence which may account for the dissimilar functional properties existing between the two types of stem cells. Compared to V-, S-ASC have a smaller surface area both in adherent and detached conditions, a more hyperpolarized RMP and a greater resting specific membrane conductance, all properties supporting a higher differentiation potential [15], [16]. These differences in electrophysiological properties are in agreement with the greater size of the noisy and rapidly activating K^+^ currents (I_BK_) and the small size of slow I_Ks_ characterizing S- versus V-ASC, which may allow S-ASC to control more efficiently the depolarization of the membrane potential induced e.g. by hormones. Accordingly, differentiated S-ADIPO compared to V-ADIPO showed a greater i) increase in cell surface, ii) reduction in the specific resting membrane conductance, iii) size reduction and faster kinetics of I_BK_ and I_Ks_ iv) increase in I_Kir_. The higher increase in surface observed in S-ADIPO, estimated by the C_m_ parameter, suggests the occurrence of a better cell growth, while the strong decrease in the specific conductance, denotes an improved membrane barrier, which represents a pivotal feature to maintain ion intracellular homeostasis. Moreover, the greater increase of I_Kir_ size together with the more hyperpolarized RMP observed in S- compared to V-ADIPO are in agreement with the reduced expression of KDR channels and the increased size of the inward-rectifier K^+^ current (I_Kir_) described as occurring during commitment and differentiation processes [14]–[17], also confirming the greater plasticity and differentiation potential of S- than V-ASC.

Finally, differentiated S-ADIPO show a more negative voltage threshold and a faster kinetic (faster time constant) of I_BK_ activation, all factors that further improve the control of membrane potential depolarization induced by external stimuli. Conversely, the minor changes observed in V-ADIPO following differentiation support the conclusion that V-ASC display a lesser differentiation potential than S-ASC. Such differences in electrophysiological properties in both stem and differentiated cells may contribute to the functional differences observed between the two populations in terms of adipokine synthesis and lipolysis. While direct correlation between proliferative/differentiation properties and electrophysiological characterization of the two stem populations is beyond the aim of the present paper, it would be of great interest to examine these parameters in parallel with ASC populations of matched SAT and VAT obtained from a larger number of subjects.

One of the main limitations of our study relates to the use of enriched stem cell populations which, although presenting very low contamination by other cell types, may respond differently in terms of proliferation and differentiation, due to the presence of non synchronized adipose precursors at different cell cycle and differentiation stages. However, cloning such cells in parallel conditions from the V- and the S- compartments is challenging and extends beyond the scope of the current work. Moreover, the non synchronized situation used in this study resembles what occurs physiologically *in vivo* within the adipose depots.

In conclusion, our results support evidence of an autonomous programming in adult adipose stem cells from SAT and VAT compartments. A greater plasticity, proliferation and differentiation potential is evident in S-ASC, which seems to be maintained along the adipose lineage and accounts for the differences observed in anatomically distinct mature adipocytes and adipose depots. VAT and SAT functional differences originate early at the level of the adult adipose stem cell which maintains its memory of the adipose tissue of origin. Such differences in the stem cell may make distinct fat pads differentially susceptible to the development of metabolic dysfunctions and may serve as targets for specific therapeutic approaches.
